# Experimental Testing, Manufacturing and Numerical Modeling of Composite and Sandwich Structures

**DOI:** 10.3390/ma17143468

**Published:** 2024-07-13

**Authors:** Raul Duarte Salgueiral Gomes Camplho

**Affiliations:** 1 CIDEM, ISEP—School of Engineering, Polytechnic of Porto, R. Dr. António Bernardino de Almeida, 431, 4200-072 Porto, Portugal; raulcampilho@gmail.com; 2 INEGI—Institute of Science and Innovation in Mechanical and Industrial Engineering, Pólo FEUP, Rua Dr. Roberto Frias, 400, 4200-465 Porto, Portugal

Composite materials have become indispensable in a multitude of industries, such as aerospace, automotive, construction, sports equipment, and electronics [1]. These materials are known for their exceptional strength-to-weight ratio, fatigue and corrosion resistance, and minimal thermal expansion. The ability to tailor composite materials to specific applications by varying the type, size, and orientation of the reinforcement material, as well as the type of matrix material used, increases their versatility and effectiveness [2]. However, issues such as high production costs, repair complexity, susceptibility to delamination and other damage types, and difficulties in characterization and modeling, are significant obstacles [3]. Currently, advanced manufacturing techniques, including additive and digital manufacturing, are being explored to reduce production costs and improve the precision and reliability of composite materials [4]. The development of nanocomposites aims to enhance strength, stiffness, thermal conductivity, and electrical conductivity, thereby broadening their application potential [5]. In parallel, sustainability demands triggered the creation of sustainable composites, which incorporate renewable or recycled materials to reduce environmental impact [6]. Multi-functional composites, integrating materials like shape memory alloys, piezoelectric materials, and carbon nanotubes, enable the creation of intelligent systems capable of self-monitoring, adaptive responses, and energy harvesting [7]. Bioinspired composites draw inspiration from natural materials, such as spider silk, seashells, and bone, striving to replicate their unique properties [8]. Numerical modeling has also seen substantial advancements, e.g., in simulating the complex behaviors of composite materials under various conditions [9].

Composite sandwich structures find application in industries such as aerospace, automotive, marine, and construction [10]. Their high strength-to-weight ratio, stiffness, and durability make them ideal for these demanding environments. Advanced materials, including fiber-reinforced polymers and metal matrix composites for face sheets, and innovative core materials, such as foams, honeycombs, and lattice structures, are being developed. Automated and robotic systems are employed to improve consistency, reduce labor costs, and enable the production of more complex geometries [11]. Advanced cores, such as 3D-printed lattice structures and bioinspired geometries, are being developed to improve impact resistance, energy absorption, and structural integrity [12]. Understanding the dynamic behavior of sandwich structures under impact, blast, and vibration is crucial [13].

Within the scope of this Special Issue, Wang et al. [14] explored the combined effects of high-current pulsed electron beam (HCPEB) treatment and CeO_2_ denaturant on enhancing the microstructure and properties of Al-20SiC composites produced by powder metallurgy. Grazing incidence X-ray diffraction (GIXRD) revealed a selective orientation of aluminum grains, particularly with Al(111) crystal faces, following HCPEB treatment.

Baimova and Shcherbinin [15] focused on three different morphologies of graphene aerogels with a honeycomb-like structure, examining their strength and deformation behavior through a molecular dynamics simulation. Their findings advanced the understanding of the microscopic deformation mechanisms in graphene aerogels.

Choi et al. [16] examined the influence of anisotropy and temperature on short carbon fiber-reinforced polyamide-6 (CF-PA6) produced via injection molding to determine its static and fatigue characteristics. The tensile strength and fatigue life of CF-PA6 varied with changes in temperature and anisotropy. A semi-empirical strain–stress fatigue life prediction model was positively validated.

A biaxial tensile test was conducted by Sanai et al. [17] to assess the fracture behavior of a unidirectional carbon fiber-reinforced plastic (CFRP) under load in the fiber (0°) and transverse (90°) directions. This study demonstrated that the occurrence of each fracture mode is solely characterized by a normal strain in the 90° direction (*ε*_y_).

In the work of Li et al. [18], an interlayer nanocomposite (CC@rGO) featuring a graphene heterojunction with CoO and Co9S8 was synthesized using a hydrothermal calcination technique and evaluated as a cathode sulfur carrier for lithium–sulfur batteries. The CC@rGO sulfur cathode exhibited excellent electrochemical performance, rate capability, and cycling stability across various current densities.

Ntaflos et al. [19] produced graphene nanoplatelet (GNP)-enhanced glass fiber-reinforced plastics (GFRP) produced by filament winding. The performance of these materials was tested under harsh environmental conditions. Results showed that GNPs improved the in-plane shear strength of GFRP by 200% and reduced water uptake by up to 40%.

Ciecieląg [20] investigated the milling of CFRP and GFRP saturated with epoxy resin using various tools (polycrystalline diamond inserts, physically coated carbide inserts with titanium nitride, and uncoated carbide inserts). Milling thin-walled CFRP with uncoated tools at high feeds per revolution and high cutting speeds resulted in the highest forces.

Knápek et al. [21] investigated how the clearance angle of milling tools affects wear, cutting forces, machined edge roughness, and delamination during the milling of CFRP panels with a twill weave and a 90° fiber orientation. The clearance angle significantly impacted tool wear, surface roughness, and the delamination of the CFRP panel. Higher tool wear led to higher cutting forces, which increased surface roughness and delamination.

Matula and Tomiczek [22] discussed the integration of surface engineering and powder metallurgy to develop coatings with enhanced corrosion resistance and wear properties. Tensile tests indicated that both steel types exhibited higher strength after sintering in a nitrogen-rich atmosphere.

Yelemessov et al. [23] experimentally and analytically explored the potential application of polymer concrete composites in building structures through a strength analysis. The rubber concrete beam deformation at failure exceeded that of cement concrete by 2.5 to 6.5 times and by 3.0 to 7.5 times with a fiber reinforcement.

Bhagatji et al. [24] addressed the fabrication, experimental testing, and progressive failure modeling of an ultra-thin composite beam. The continuum damage mechanics (CDM) model for the beam, calibrated through experimental coupon testing, was utilized in finite element explicit analysis. The finite element model accurately predicted localized transverse fiber damage under eccentric buckling.

Kwon et al. [25] investigated specimens made from a variety of materials with different geometric features to predict failure loads using a newly proposed criterion that incorporates both stress and stress gradient conditions. The types of notches examined included cracks and holes. There was a good agreement between experimental data and theoretical predictions across all cases.

The work of Ferreira et al. [26] introduced an efficient method to study the low-velocity impact response of woven composite shells using 3D finite element models that incorporate both intralaminar and interlaminar progressive damage mechanisms. The numerical predictions closely matched experimental data regarding load and energy histories, maximum impact load, displacement, and contact time.

Kraisornkachit et al. [27] investigated adhesive joint strength and elastic modulus, critical for adhesive performance. The experiments employed a bisphenol A-based epoxy resin with a polypropylene glycol curing agent, generating initial data from 32 conditions used to train a machine learning model. Bayesian optimization identified conditions surpassing this boundary, achieving an adhesive joint strength of 25.2 MPa and an elastic modulus of 182.5 MPa.

Takamura et al. [28] performed a numerical analysis on single-lap shear joints between carbon fiber-reinforced thermoplastics (CFRTPs) to estimate the local stress state at the point of failure initiation, revealing the true bonding strength. Results indicated that the single-lap shear test underestimates the apparent bonding strength by less than 14% of the true bonding strength.

Khademi et al. [29] studied a material system comprising unidirectional carbon fiber composite face sheets with a honeycomb core, investigating various defects at this critical interface. The work introduced high-frequency ultrasound testing (UT) to detect and quantify the geometry and type of defects. The results indicated successful defect detection.

Wang et al. [30] enhanced sound insulation in composite structures through strategic material arrangement. Initially, a predictive model for sound insulation in sandwich composite plates was developed and validated. Optimization strategies for sound insulation in high-speed train composite floors were explored. Implementing this approach in high-speed train body design improved sound insulation by 1–3 dB in the 125–315 Hz band.

The study of Grzybek [31] involved experimental analysis of the control systems for piezoelectric actuators based on composite and aluminum materials, specifically examining unimorph and bimorph structures. Two piezoelectric actuators were manufactured with a cantilever sandwich beam configuration: one with a glass-reinforced epoxy composite (FR4) carrier layer and another with 1050 aluminum. The study also proposed a modification to the linear quadratic regulator (LQR) control algorithm.

Barbinta-Patrascu et al. [32] explored a sustainable approach using weeds to produce silver nanoparticles (AgNPs) with diverse potential applications. Two different types of AgNPs were generated by varying the ratio of phytoextract to silver salt solution. The resulting green composite materials were characterized and showed significant potential for designing innovative bioactive materials suitable for biomedical applications.

Grząbka-Zasadzińska et al. [33] investigated the potential of using caffeine-treated and untreated black cherry (Prunus serotina Ehrh.) wood as a filler in polylactide composites. The study highlighted the novel application of caffeine as a natural compound to modify wood, thereby altering the supermolecular structure and nucleating abilities of polylactide/wood composites.

Taking into account all of the collected contributions, the future of research in experimental testing, manufacturing, and numerical modeling of composite and sandwich structures is expected to advance towards more integrated, multi-functional, and sustainable solutions that address diverse industry needs through innovations in material science and engineering. The identified hot topics are:

- Multi-Material and Hybrid Structures: Combining different materials (composites with metals, ceramics, and polymers) to achieve superior performance in, for example, enhanced mechanical properties, tailored thermal and electrical conductivity, and improved damage tolerance [34].
- Advanced Manufacturing Techniques: Additive manufacturing (3D printing), automated layup methods (such as automated fiber placement and tape laying), and innovative curing techniques to enhance production efficiency and reduce costs [35].
- Durability and Environmental Resistance: Developing coatings, surface treatments, and materials that resist degradation from environmental factors (ultraviolet or UV exposure, moisture, and chemicals) and promote an extended service life [36].
- Damage Detection and Structural Health Monitoring: Integrating embedded sensors, data analytics, and machine learning algorithms to enable real-time monitoring and predictive maintenance of composite structures [37].
- Simulation and Modeling: Developing multi-scale models that capture the complex behavior of composites, from microstructure to macroscopic performance, and validating these models with experimental data [38].
- Functional and Smart Materials: Integration of functional materials (such as shape memory alloys, piezoelectric materials, and sensors) into sandwich structures to exhibit adaptive, self-healing, or sensing capabilities [39].
- Sustainability and Recycling: Developing eco-friendly manufacturing processes, bio-based resins, and recyclable composite and sandwich structures. Improving recycling methods to recover and reuse materials effectively [40].
